# Phthalimide analogs as probable 15-lipoxygenase-1 inhibitors: synthesis, biological evaluation and docking studies

**DOI:** 10.1186/s40199-015-0118-5

**Published:** 2015-07-22

**Authors:** Alireza Aliabadi, Ahmad Mohammadi-Farani, Zeinab Hosseinzadeh, Hamid Nadri, Alireza Moradi, Farahnaz Ahmadi

**Affiliations:** Pharmaceutical Sciences Research Center, School of Pharmacy, Kermanshah University of Medical Sciences, Kermanshah, Iran; Department of Medicinal Chemistry, Faculty of Pharmacy, Kermanshah University of Medical Sciences, Kermanshah, Iran; Department of Pharmacology, Toxicology and Medical Services, Faculty of Pharmacy, Kermanshah University of Medical Sciences, Kermanshah, Iran; Students Research Committee, Kermanshah University of Medical Sciences, Kermanshah, Iran; Neurobiomedical Research Center, Yazd Shahid Sadoughi University of Medical Sciences, Yazd, Iran

**Keywords:** Synthesis, Phthalimide, 1,3,4-Thiadiazole, Lipoxygenase, Anticancer

## Abstract

**Background:**

Recent studies have been explained the role of lipoxygenases (LOX) in the origin of cancer. Among the lipoxygenases, the 5-LOX, 12-LOX and 15-LOX are more important in the cause of neoplastic disorders. In the present investigation, a new series of anticancer agents with 1,3,4-thiadiazole and phthalimide substructures were synthesized and their *in vitro* cytotoxicity was evaluated by MTT assay. Moreover, enzyme inhibitory potency was also assessed by enzymatic protocol towards 15-LOX-1. Molecular docking was performed to explore *in silico* binding mode of the target compounds.

**Results:**

Tested compounds showed a better cytotoxic activity against HT29 cell line (colorectal cancer) in comparison with other cell lines (PC3: prostate carcinoma; SKNMC: neuroblastoma). Unfortunately, all of the tested derivatives rendered lower inhibitory potency than quercetin towards 15-LOX-1. Four hydrogen bonds were detected in docking studies for compound **4d** as the most potent derivative in enzymatic assay.

**Conclusions:**

The biological results of reported compounds in this research were not so satisfactory. But, further structural modifications are necessary to improve the bioactivity of these derivatives.

## Background

Arachidonic acid is a fatty acid released from membrane phospholipids during cell stimulation. Arachidonic acid is metabolized mainly by two groups of enzymes consist of lipoxygenases (LOX, which includes 5-LOX, 12-LOX, and 15-LOX) and cyclooxygenase (COX). Inhibition of cyclooxygenases delays tumorigenesis in animals and humans [1, 2]. Various epidemiological and animal studies have confirmed that there is a close relationship between high fat consumption with an increased incidence and growth of tumors at several specific organ sites like breast. More recent studies also presented that patients with consumption of diets with a high proportion of polyunsaturated ω-6 fatty acid (n-6 PUFA), such as arachidonic acid and linoleic acid are associated with a more advanced disease stage at the time of diagnosis of breast cancer [2]. The LOXs convert polyunsaturated fatty acids like arachidonic and linoleic acids into biologically active metabolites that affects various cellular events such as signaling, structure and metabolism. According to the later tumorigenesis studies, it is likely that polyunsaturated fatty acids may enhance tumorigenesis via oxidative metabolism. Eicosanoids derived from the arachidonic acid cascade have been implicated in the pathogenesis of a variety of human diseases, including cancer, and are now believed to play important roles in tumor promotion, progression, and metastatic disease [3–5]. Vigorous expression of the enzymes (LOXs & COXs) that synthesize bioactive lipid metabolites from unsaturated fatty acids (arachidonic acid and linoleic acid) appears to be related to the development of prostate carcinoma remarkably. Other research have also reported that 15-LO-2 is expressed in normal prostate tissue, but poorly expressed in prostate tumors. Thus, 15-LO-1 is highly expressed in prostate tumors while 15-LO-2 is highly expressed in normal tissue. 15-LO-1 in prostate cancer tumors converts linoleic acid, its preferred substrate to 13-*S*-hydroxy-octadecadienoic acid (13-(*S*)-HODE) and other metabolites. These metabolites appear to alter cellular signaling pathways, and thus the inappropriate expression might alter biological events and contribute to tumor development [6–8]. On the basis of this information, the drugs with capability of interaction with pathways related to the production of lipoxygenases metabolites or signaling functions of lipoxygenases products may be effective pharmaceutical agents in prevention or treatment of cancer.

**Scheme 1:**
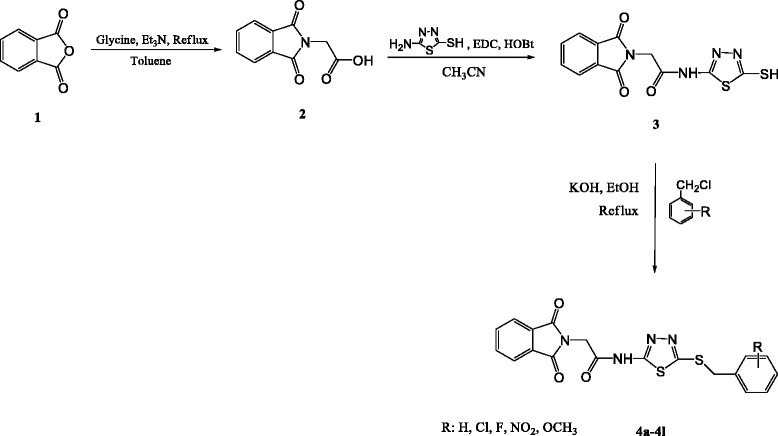
Synthetic pathway for preparation of compounds 4a-4 l

Literature reviews declare that 1,3,4-thiadiazole ring as 5-membered heterocycle have diverse biological effects such as anti-inflammatory, anticonvulsant, antibacterial, antileshmanial, antioxidant and anticancer [9–20]. On the other hands, phthalimide moiety is another heterocyclic residue derived from the isoindole ring. This moiety has also exhibited several pharmacological activities like anticonvulsant, antitubercular, anti-inflammatory, anti-acetylcholinesterase as well as anticancer effects [21–31]. In the current investigation, we encouraged to synthesize a new series of chemical entities bearing 1,3,4-thiadiazole and phthalimide (isoindoline-1,3-dione) residues as potential anticancer agents.

## Methods

### Chemistry

The corresponding chemical reagents and starting materials were purchased from the commercial companies such as Merck and Sigma-Aldrich. The purification of the prepared compounds was carried out by column chromatography using ethyl acetate/petroleum ether. Spectroscopic methods were applied for characterization of the synthesized compounds. ^1^H NMR spectra were acquired by Bruker 250 MHz in deutrated chloroform (CDCl_3_) and the obtained data were expressed as δ (ppm) compared to tetramethylsilane (TMS) as internal standard. Infrared (IR) spectra of the prepared compounds were obtained by Shimadzu 470 with preparing potassium bromide (KBr) disk. The mass spectra were run on a Finigan TSQ-70 spectrometer (Finigan, USA) at 70 eV. Melting points were determined using electrothermal 9001 melting point analyzer apparatus and are uncorrected.

### *Synthesis of 2-(1,3-Dioxoisoindolin-2-yl)acetic acid* (2)

5 g (33.8 mmol) of phthalic anhydride, 2.53 g (33.8 mmol) glycine and 4.67 ml (33.8 mmol) triethylamine (Et_3_N) were mixed in toluene (100 ml) and the reaction mixture was refluxed overnight (Scheme 1). The reaction was monitored by thin layer chromatography (TLC). Toluene was evaporated by rotary evaporator apparatus under reduced pressure. The obtained residue was washed by diethyl ether (Et_2_O) and *n*-hexane. The obtained white powder was used for the next step without any extra purification [32].

^1^HNMR (CDCl_3_, 250 MHz) δ (ppm): 4.3 (s, 2H, −CH_2_-), 7.70 (dd, 2H, *J* = 8.4 Hz, *J* = 2.4 Hz, H_5,6_-Phthalimide), 7.85 (dd, 2H, *J* = 8.4 Hz, *J* = 2.4 Hz, H_4,7_-Phthalimide), 11.97 (brs, −COOH). IR (KBr, cm^−1^) ῡ: 3468 (OH, acid), 3155, 2989, 2939, 1705 (C = O, acid). MS (*m/z*, %): 205 (M^+^, weak), 160 (100), 133 (20), 104 (40), 76 (35), 50 (20).

### *Synthesis of 2-(1,3-Dioxoisoindolin-2-yl)-N-(5-mercapto-1,3,4-thiadiazol-2-yl)acetamide* (3)

3 g (14.63 mmol) of 2-(1,3-dioxoisoindolin-2-yl)acetic acid (compound **2**), 2.80 g (14.63 mmol) *N*-ethyl-*N*-dimethylaminopropyl carbodiimide (EDC) and 1.98 g (14.63 mmol) hydroxybenzotriazole (HOBt) were mixed in 70 ml acetonitrile (CH_3_CN) and the obtained mixture was stirred for 30 min. Then, 1.95 g (14.63 mmol) of 5-amino-1,3,4-thiadiazole-2-thiol was added to the reaction mixture and the stirring was continued for 24 h. The reaction end was proved by thin layer chromatography (TLC). Then, acetonitrile was evaporated using rotary evaporator and the residue was washed by diethyl ether and *n*-hexane. More purification was done by column chromatogaraphy (petroleum ether/ethyl acetate 70/30) to afford a yellowish powder [33–35].

^1^HNMR (CDCl_3_, 250 MHz) δ (ppm): 4.81 (s, 2H, −CH_2_-CO-), 3.82 (s, 1H, SH), 7.89 (dd, 2H, *J* = 8.4 Hz, *J* = 2.4 Hz, H_5,6_-Phthalimide), 7.94 (dd, 2H, *J* = 8.4 Hz, *J* = 2.4 Hz, H_5,6_-Phthalimide), 13.81 (brs, NH). IR (KBr, cm^−1^) ῡ: 3201, 3070, 2924, 1739, 1608, 1554, 1465, 1377, 1307, 1053, 717. MS (*m/z*, %): 320 (10), 287 (80), 263 (60), 231 (100), 160 (80), 159 (60), 121 (40), 104 (40), 76 (45).

### *General procedure for synthesis of compounds* 4a-4 l

In a flat bottom flask, 0.2 g (0.625 mmol) of 2-(1,3-dioxoisoindolin-2-yl)-*N*-(5-mercapto-1,3,4-thiadiazol-2-yl)acetamide (compound **3**) was treated with 0.035 g (0.625 mmol) potassium hydroxide in absolute ethanol and heated for 5 min, then equimolar (0.625 mmol) quantity of appropriate benzyl chloride derivative was added to the reaction medium and reflux condition was performed for 24 h. Thin layer chromatography (TLC) was carried out to determine the reaction end. Then, crushed ice was added to the reaction flask and the formed precipitate was filtered and collected. Crystallization was performed using ethanol [35].

### *2-(1,3-Dioxoisoindolin-2-yl)-N-(5-(2-nitrobenzylthio)-1,3,4-thiadiazol-2-yl)acetamide* (4a)

^1^HNMR (CDCl_3_, 250 MHz) δ (ppm): 4.79 (s, 2H, −S-CH_2_-), 4.89 (s, 2H, −CH_2_-CO-), 7.45 (t, 1H, H_4_-2-Nitrophenyl), 7.57 (m, 2H, H_5,6_-2-Nitrophenyl), 7.81 (dd, 2H, *J* = 8.4 Hz, *J* = 2.4 Hz, H_5,6_-Phthalimide), 7.95 (dd, 2H, *J* = 8.4 Hz, *J* = 2.4 Hz, H_5,6_-Phthalimide), 8.06 (d, 1H, *J* = 10 Hz, H_3_-2-Nitrophenyl). IR (KBr, cm^−1^) ῡ: 3414, 3325, 3170, 3035, 2927, 2854, 2742, 1774, 1720, 1573, 1523, 1415, 1346, 1303, 1195, 952, 713.

### *2-(1,3-Dioxoisoindolin-2-yl)-N-(5-(3-nitrobenzylthio)-1,3,4-thiadiazol-2-yl)acetamide* (4b)

^1^HNMR (CDCl_3_, 250 MHz) δ (ppm): 4.45 (s, 2H, −S-CH_2_-), 4.77 (s, 2H, −CH_2_-CO-), 7.48-7.59 (m, 2H, 3-Nitrophenyl), 7.73 (dd, 2H, *J* = 8.4 Hz, *J* = 2.4 Hz, H_5,6_-Phthalimide), 7.92 (d, 1H, *J* = 7.5 Hz, H_4_-3-Nitrophenyl), 8.24 (dd, 2H, *J* = 8.4 Hz, *J* = 2.4 Hz, H_4,7_-Phthalimide), 8.36 (s, 1H, H_2_-3-Nitrophenyl). IR (KBr, cm^−1^) ῡ: 3429, 3209, 3066, 2924, 2854, 1774, 1708, 1527, 1411, 1350, 1296, 1195, 1053, 952, 717. MS (*m/z*, %): 455 (M^+^, Weak), 398 (15), 268 (100), 235 (35), 193 (45), 136 (55), 109 (15), 89 (30), 60 (35).

### *2-(1,3-Dioxoisoindolin-2-yl)-N-(5-(4-nitrobenzylthio)-1,3,4-thiadiazol-2-yl)acetamide* (4c)

^1^HNMR (CDCl_3_, 250 MHz) δ (ppm): 4.42 (s, 2H, −S-CH_2_-), 4.79 (s, 2H, −CH_2_-CO-), 7.52 (d, 2H, *J* = 10 Hz, H_2,6_-4-Nitrophenyl), 7.79 (dd, 2H, *J* = 8.4 Hz, *J* = 2.4 Hz, H_5,6_-Phthalimide), 7.91 (dd, 2H, *J* = 8.4 Hz, *J* = 2.4 Hz, H_4,7_-Phthalimide), 8.2 (d, 2H, *J* = 10 Hz, H_2,6_-4-Nitrophenyl). IR (KBr, cm^−1^) ῡ: 3421, 3352, 3113, 2927, 2850, 1774, 1720, 1519, 1415, 1346, 1303, 1107, 952, 713. MS (*m/z*, %): 455 (M^+^, Weak), 398 (12), 268 (100), 235 (20), 193 (90), 136 (30), 109 (25), 89 (60), 60 (40).

### *2-(1,3-Dioxoisoindolin-2-yl)-N-(5-(3-methoxybenzylthio)-1,3,4-thiadiazol-2-yl)acetamide* (4d)

^1^HNMR (CDCl_3_, 250 MHz) δ (ppm): 3.74 (s, 3H, −OCH_3_), 4.38 (s, 2H, −S-CH_2_-), 4.86 (s, 2H, −CH_2_-CO-), 6.77 (d, 1H, *J* = 10 Hz, H_4_-3-Methoxyphenyl), 6.92 (m, 2H, H_2_, H_6_ -3-Methoxyphenyl), 7.20 (t, 1H, *J* = 7.5 Hz, H_5_-3-Methoxyphenyl), 7.77 (dd, 2H, *J* = 8.4 Hz, *J* = 2.4 Hz, H_5,6_-Phthalimide), 7.92 (dd, 2H, *J* = 8.4 Hz, *J* = 2.4 Hz, H_4,7_-Phthalimide). IR (KBr, cm^−1^) ῡ: 3425, 3329, 3170, 3035, 2927, 2850, 1720, 1624, 1577, 1415, 1303, 1269, 1159, 1049, 952, 713. MS (*m/z*, %): 440 (M^+^, 10), 160 (40), 121 (100), 104 (45), 92 (20), 76 (35), 65 (60), 52 (55).

### *2-(1,3-Dioxoisoindolin-2-yl)-N-(5-(4-methoxybenzylthio)-1,3,4-thiadiazol-2-yl)acetamide* (4e)

^1^HNMR (CDCl_3_, 250 MHz) δ (ppm): 3.76 (s, 3H, −OCH_3_), 4.35 (s, 2H, −S-CH_2_-), 4.85 (s, 2H, −CH_2_-CO-), 6.82 (d, 2H, *J* = 7.5 Hz, H_3,5_-4-Mehoxyphenyl), 7.28 (d, 2H, *J* = 7.5 Hz, H_2,6_-4-Mehoxyphenyl), 7.77 (dd, *J* = 8.4 Hz, *J* = 2.4 Hz, 2H, H_5,6_-Phthalimide), 7.92 (dd, 2H, *J* = 8.4 Hz, *J* = 2.4 Hz, H_5,6_-Phthalimide). IR (KBr, cm^−1^) ῡ: 3325, 3248, 3132, 3035, 2927, 2850, 1774, 1705, 1627, 1577, 1419, 1307, 1246, 1195, 1180, 1064, 952. MS (*m/z*, %): 440 (M^+^, Weak), 249 (10), 193 (10), 160 (60), 121 (100), 104 (30), 92 (40), 76 (75), 65 (70), 52 (45).

### *2-(1,3-Dioxoisoindolin-2-yl)-N-(5-(2-fluorobenzylthio)-1,3,4-thiadiazol-2-yl)acetamide* (4f)

^1^HNMR (CDCl_3_, 250 MHz) δ (ppm): 4.47 (s, 2H, S-CH_2_-), 4.83 (s, −CH_2_-CO-), 7.04-7.09 (m, 4H, 2-Fluorophenyl), 7.79 (dd, 2H, *J* = 8.4 Hz, *J* = 2.4 Hz, H_5,6_-Phthalimide), 7.91 (dd, 2H, *J* = 8.4 Hz, *J* = 2.4 Hz, H_4,7_-Phthalimide). IR (KBr, cm^−1^) ῡ: 3429, 3329, 3170, 2924, 2854, 2738, 1774, 1720, 1624, 1573, 1492, 1415, 1303, 1195, 952, 759. MS (*m/z*, %): 428 (M^+^, 12), 371 (25), 241 (20), 166 (30), 109 (100), 83 (10).

### *2-(1,3-Dioxoisoindolin-2-yl)-N-(5-(3-fluorobenzylthio)-1,3,4-thiadiazol-2-yl)acetamide* (4 g)

^1^HNMR (CDCl_3_, 250 MHz) δ (ppm): 4.39 (s, 2H, −S-CH_2_-), 4.85 (s, 2H, −CH_2_-CO-), 6.92 (t, 1H, *J* = 7.5 Hz, H_5_-3-Fluorophenyl), 7.11 (m, 2H, H_4_, H_6_ -3-Fluorophenyl), 7.24 (m, 1H, H_2_-3-Fluorophenyl), 7.77 (dd, 2H, *J* = 8.4 Hz, *J* = 2.4 Hz, H_5,6_-Phthalimide), 7.92 (dd, 2H, *J* = 8.4 Hz, *J* = 2.4 Hz, H_5,6_-Phthalimide), 13.70 (brs, NH). IR (KBr, cm^−1^) ῡ: 3444, 3170, 3035, 2924, 2854, 1774, 1720, 1566, 1415, 1300, 952, 713. MS (*m/z*, %): 428 (M^+^, 15), 371 (30), 241 (40), 166 (25), 109 (100), 83 (10).

### *2-(1,3-Dioxoisoindolin-2-yl)-N-(5-(4-fluorobenzylthio)-1,3,4-thiadiazol-2-yl)acetamide* (4 h)

^1^HNMR (CDCl_3_, 250 MHz) δ (ppm): 4.36 (s, 2H, −S-CH_2_-), 4.86 (s, 2H, −CH_2_-CO-), 6.99 (t, 2H, H_2,6_-4-Fluorophenyl), 7.32 (t, 2H, H_3,5_-4-Fluorophenyl), 7.77 (dd, 2H, *J* = 8.4 Hz, *J* = 2.4 Hz, H_5,6_-Phthalimide), 7.92 (dd, 2H, *J* = 8.4 Hz, *J* = 2.4 Hz, H_4,7_-Phthalimide). IR (KBr, cm^−1^) ῡ: 3433, 3329, 3159, 3039, 2927, 2850, 1774, 1720, 1624, 1573, 1508, 1415, 1303, 1226, 1087, 952, 831, 713. MS (*m/z*, %): 428 (10), 371 (20), 241 (10), 166 (40), 109 (100), 83 (12).

### *N-(5-(2-Chlorobenzylthio)-1,3,4-thiadiazol-2-yl)-2-(1,3-dioxoisoindolin-2-yl)acetamide* (4i)

^1^HNMR (CDCl_3_, 250 MHz) δ (ppm): 4.54 (s, 2H, −S-CH_2_-), 4.87 (s, 2H, −CH_2_-CO-), 7.20 (m, 2H, H_3,6_-2-Chlorophenyl), 7.35 (t, 1H, H_4_-2-Chlorophenyl), 7.43 (t, 1H, H_5_-2-Chlorophenyl), 7.77 (dd, 2H, *J* = 8.4 Hz, *J* = 2.4 Hz, H_5,6_-Phthalimide), 7.92 (dd, 2H, *J* = 8.4 Hz, *J* = 2.4 Hz, H_4,7_-Phthalimide). IR (KBr, cm^−1^) ῡ: 3475, 3429, 3170, 3051, 2927, 2858, 2738, 1774, 1720, 1566, 1469, 1415, 1300, 1195, 952, 713. MS (*m/z*, %): 446 (M^+^+2, 4), 444 (M^+^, 10), 284 (12), 162 (10), 160 (85), 125 (100), 104 (10), 76 (15).

### *N-(5-(3-Chlorobenzylthio)-1,3,4-thiadiazol-2-yl)-2-(1,3-dioxoisoindolin-2-yl)acetamide* (4j)

^1^HNMR (CDCl_3_, 250 MHz) δ (ppm): 4.37 (s, 2H, −S-CH_2_-), 4.85 (s, 2H, −CH_2_-CO-), 7.24 (m, 3H, 3-Chlorophenyl), 7.41 (s, 1H, H_2_-3-Chlorophenyl), 7.77 (dd, 2H, *J* = 8.4 Hz, *J* = 2.4 Hz, H_5,6_-Phthalimide), 7.92 (dd, 2H, *J* = 8.4 Hz, *J* = 2.4 Hz, H_4,7_-Phthalimide). IR (KBr, cm^−1^) ῡ: 3325, 3170, 3055, 2927, 2850, 1720, 1627, 1573, 1400, 1300, 1203, 1111, 1083, 1049, 956, 713, 648. MS (*m/z*, %): 446 (M^+^+2, 5), 444 (M^+^, 15), 162 (25), 160 (50), 125 (100), 104 (30), 76 (35).

### *N-(5-(4-Chlorobenzylthio)-1,3,4-thiadiazol-2-yl)-2-(1,3-dioxoisoindolin-2-yl)acetamide* (4 k)

^1^HNMR (CDCl_3_, 250 MHz) δ (ppm): 4.35 (s, 2H, −S-CH_2_-), 4.85 (s, 2H, −CH_2_-CO-), 7.28 (dd, 4H, 4-Chlorophenyl), 7.77 (dd, 2H, *J* = 8.4 Hz, *J* = 2.4 Hz, H_5,6_-Phthalimide), 7.91 (dd, 2H, *J* = 8.4 Hz, *J* = 2.4 Hz, H_5,6_-Phthalimide). IR (KBr, cm^−1^) ῡ: 3325, 3155, 3059, 3035, 2927, 2850, 1774, 1720, 1627, 1573, 1492, 1303, 1091. MS (*m/z*, %): 446 (M^+^+2, 5), 444 (M^+^, 12), 284 (5), 162 (15), 160 (90), 125 (100), 104 (20), 76 (25).

### *N-(5-(Benzylthio)-1,3,4-thiadiazol-2-yl)-2-(1,3-dioxoisoindolin-2-yl)acetamide* (4 l)

^1^HNMR (CDCl_3_, 250 MHz) δ (ppm): 4.39 (s, 2H, −S-CH_2_-), 4.86 (s, 2H, −CH_2_-CO-), 7.23-7.37 (m, 5H, Phenyl), 7.77 (dd, 2H, *J* = 8.4 Hz, *J* = 2.4 Hz, H_5,6_-Phthalimide), 7.92 (dd, 2H, *J* = 8.4 Hz, *J* = 2.4 Hz, H_4,7_-Phthalimde). IR (KBr, cm^−1^) ῡ: 3425, 3329, 3170, 3032, 2927, 2850, 1720, 1627, 1573, 1546, 1415, 1396, 1303, 1195, 1111, 952, 709. MS (410, M^+^), 160 (75), 91 (100), 77 (10).

### MTT assay

Synthesized derivatives (compounds **4a-4 l**) were tested for cytotoxic activity at 0.1-100 μM concentration in three human cancer cell lines of PC3 (Prostate carcinoma), HT29 (Colorectal cancer) and SKNMC (Neuroblastoma). Cells were purchased from the Pasteur Institute of Iran. Cells from different cell lines were seeded in 96-well plates at the density of 8000–10,000 viable cells per well and incubated for 24 h to allow cell attachment. The cells were then incubated for another 24 h with various concentrations of compounds **4a-4 l**. Cells were then washed in PBS, and 20 μL of MTT (3–(4,5-dimethylthiazol-2-yl)-2,5-diphenyl tetrazolium bromide solution (5 mg/mL) were added to each well. An additional 4 h of incubation at 37 °C were done, and then the medium was discarded. Dimethyl sulfoxide (60 μL) was added to each well, and the solution was vigorously mixed to dissolve the purple tetrazolium crystals. The absorbance of each well was measured by plate reader (Anthous 2020; Austria) at a test wavelength of 550 nm against a standard reference solution at 690 nm. The amount of produced purple formazan is proportional to the percentage of cell viability [33–35].

### 15-Lipoxygenase-1 assay

The basis of this method is oxidative coupling of 3-methyl-2-benzothiazolinone hydrazone (MBTH) with 3 (dimethylamino) benzoic acid (DMAB) in a hemoglobin catalyzed reaction. This reaction is initiated in the presence of lipoxygenase reaction product, linoleic acid hydroperoxide and results in a blue color formation which has a peak absorbtion at 590 nm [36]. Quercetin was used as the reference compound. Linoleic acid and two stock solutions (A and B) were prepared first. Solution A contained 50 mM DMAB and l00 mM phosphate buffer (pH = 7.0). Solution B was prepared by mixing 10 mM MBTH (3 mL) and hemoglobin (5 mg/mL, 3 mL) in 50 mM phosphate buffer at pH 5.0 (25 mL). A linoleic acid solution (1 mg/ml) was prepared by diluting 5 mg linoleic acid (solubilised in 0.5 ml ethanol) with KOH 100 mM.

For each compound the samples were solved in ethanol (25 μl) and mixed in a test tube with SLO (4000 units/mL, prepared in 50 mM phosphate buffer pH = 7.0, 25 μL) and phosphate buffer (50 mM, pH = 7, 900 μL). After a 5 min delay at room temperature, 50 μL linoleic acid was added to the mixture to start the hydroperoxidation reaction. After 8 min, solution A (270 μL) and solution B (130 μL) were added to the above mixture. 5 min later, 200 μL of SDS solution (2 %) was added to stop the reaction. The absorbance at 590 nm was compared with control (ethanol without sample).

### Docking

The related protein structure was downloaded from brookhaven protein data bank (http://RCSB.org). Namely, 15-Lipoxygenase-1 in complex with dihydroxybenzoic acid (pdb code: 1N8Q) was utilized. ArgusLab software 4.0 was used for drawing the chemical structures and then energy minimization was carried out using AM1 as semiemperical method [37]. The related ligand groups as well as binding site groups were defined. The binding location of dihydroxybenzoic aicd was defined as binding site for searching the best pose and conformation for all ligands. The geometry optimization of the protein structure was done by universal force field (UFF) as molecular mechanic method. Binding mode and related interactions of ligands with lipoxygense enzyme were explored in ArgusLab software.

## Results and discussion

### Chemistry

According to the Table 1 all intermediate and final compounds were prepared with an average yield. Compound **4a** with *ortho* nitro moiety obtained with a low yield (39 %) and compound **4c** with *para* nitro moiety prepared with an acceptable yield (74 %). For affording compound **2**, phthalic anhydride was reacted with glycine in the presence of triethylamine in toluene under reflux conditions to perform a Gabriel reaction. The white powder of compound **2** was treated with *N*-ethyl-*N*-dimethylaminopropyl carbodiimide (EDC) and hydroxybenzotriazole (HOBt) in acetonitrile and after 30 min, 5-amino-1,3,4-thiadiazole-2-thiol was added to form an amidic bond. The obtained thiol derivative was used for synthesis of the final products **4a–4 l** in alkaline medium that generated by potassium hydroxide in refluxing ethanol.

**Table 1:** Properties of synthesized compounds


Compound	(R)	Closed formula	MW (g/mol)	m.p (°C)	Yield (%)
2	-	C_10_H_7_NO_4_	205.4	115	70
3	-	C_12_H_8_N_4_O_3_S_2_	320.3	220	48
4a	*o*-NO_2_	C_19_H_13_N_5_O_5_S_2_	455.4	179	39
4b	*m*-NO_2_	C_19_H_13_N_5_O_5_S_2_	455.4	243	46
4c	*p*-NO_2_	C_19_H_13_N_5_O_5_S_2_	455.4	204	74
4d	*m*-OCH_3_	C_20_H_16_N_4_O_4_S_2_	440.5	186	43
4e	*p*-OCH_3_	C_20_H_16_N_4_O_4_S_2_	440.5	259	41
4f	*o*-F	C_19_H_13_FN_4_O_3_S_2_	428.4	190	48
4 g	*m*-F	C_19_H_13_FN_4_O_3_S_2_	428.4	173	40
4 h	*p*-F	C_19_H_13_FN_4_O_3_S_2_	428.4	154	57
4i	*o*-Cl	C_19_H_13_ClN_4_O_3_S_2_	444.9	205	47
4j	*m*-Cl	C_19_H_13_ClN_4_O_3_S_2_	444.9	188	57
4 k	*p*-Cl	C_19_H_13_ClN_4_O_3_S_2_	444.9	155	45
4 l	H	C_19_H_14_N_4_O_3_S_2_	410.4	187	53

Synthesized compounds were characterized by spectroscopic methods such as ^1^H NMR, IR and MS and corresponding melting points were also measured. Compound **4a** with *ortho* nitro moiety rendered the lowest melting point (179 °C) among the final products and compounds **4e** with *para* methoxy group demonstrated the highest melting point (259 °C) in these series. ^1^H NMR spectra were acquired in deutrated chloroform (CDCl_3_). In the most cases the acidic property of the proton of the amidic bond (NH group) was caused to not be appeared in the NMR spectra. Phthalimide group as well as 1,3,4-thiadiazole ring function as electron withdrawing groups and these have an important role in enhancing the acidic property of the hydrogen of NH group.

### Cytotoxicity evaluation

Three cancerous cell lines were used to test the anticancer activity of the final compounds **4a-4 l**. PC3 (Prostate carcinoma), HT29 (colorectal cancer) and SKNMC (neuroblastoma) was cultured and intended derivatives were assessed at concentration 0.1-100 μM and the obtained results were compared to doxorubicin as reference drug. None of the tested compounds showed superior cytotoxic effect than doxorubicin at tested concentrations towards the utilized cell lines. Generally tested derivatives exerted a better cytotoxic activity against HT29 cell line compared to other cell lines. PC3 and SKNMC cell lines were the most resistant cell lines to the tested compounds. None of the introduced moieties containing electron withdrawing groups and electron donating groups on the phenyl residue were efficacious to produce a remarkable anticancer activity.

### Enzymatic assay

An enzyme inhibitory assay was performed towards 15-lipoxygenase-1 and obtained results were presented as percent of inhibition and provided in Table 2. Unfortunately, none of the tested derivatives demonstrated superior inhibitory effect than quercetin as reference compound and natural product inhibitor of the enzyme. Compound **4d** with *meta* positioning of the methoxy moiety was the most potent inhibitor in this series (38 % inhibition). Moving the position of the methoxy to the *para* decreased the inhibitory effect of the compound significantly as observed in compound **4e**. Nitro containing derivatives (**4a, 4b, 4c**) and compound **4 k** with *para* positioning of the chlorine substituent did not show any inhibitory activity against 15-lipoxygenase-1.

**Table 2:** Biological data of synthesized compounds. Results of the cytotoxicity assay were reported as IC_50_ ± SD (μM)

Compound	R	15-Lipoxygenase-1 (% of inhibition)^a^	PC3	HT29	SKNMC
4a	*o*-NO_2_	NA^b^	100<	100<	100<
4b	*m*-NO_2_	NA	>100	100<	100<
4c	*p*-NO_2_	NA	100<	100<	100<
4d	*m*-OCH_3_	38	100<	100<	100<
4e	*p*-OCH_3_	11	100<	100<	100<
4f	*o*-F	31	100<	10.91 ± 4.1	100<
4 g	*m*-F	26	100<	100<	50.2 ± 5.4
4 h	*p*-F	35	88.83 ± 4.3	100<	100<
4i	*o*-Cl	17	81.92 ± 4.7	100<	100<
4j	*m*-Cl	18.5	89.21 ± 5.7	100<	100<
4 k	*p*-Cl	NA^a^	>100	24.06 ± 3.1	69.7 ± 3.6
4 l	H	19	>100	100<	100<
Doxorubicin	-	-	3.8 ± 0.75	2.1 ± 0.26	1.3 ± 0.4
Quercetin	-	100	-	-	-

a:The percent of inhibition was determined at 200 μM concentration

b:No activity

### Molecular modeling

**Fig. 1:**
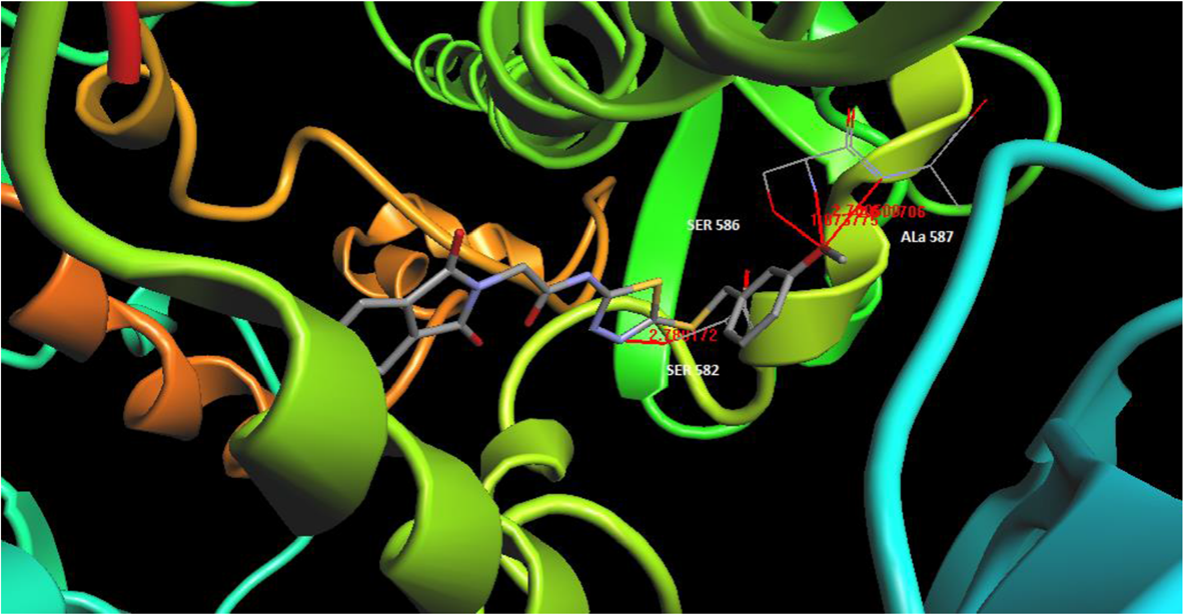
Structure of docked compound 4d (*m*-OCH_3_) into the active site of 15-lipoxygenase. Four hydrogen bonds were detected with Ser 582 (one), Ser 586 (two) and Ala 587 (one)

All prepared derivatives were docked by ArgusLab software into the active site of 15-lipoxygenase-1. Compound **4d** (*m*-OCH_3_) as representative of synthesized compounds in this series that showed a superior enzyme inhibitory activity in enzymatic assay has been shown in Fig. 1. Four hydrogen bonds were detected with Ser 582, Ser 586 and Ala 587. One of the nitrogen atom of the 1,3,4-thiadiazole ring has participated in hydrogen bonding interaction with Ser 582. Amino acid Ser 586 has also formed two hydrogen bonding interactions with oxygen of the methoxy group. The first one is with oxygen atom of the hydroxyl group in the side chain of this amino acid and the second one is with related NH_2_ group of the Ser 586. Finally, Ala 587 is responsible for the fourth hydrogen bond interaction. The NH of the amidic bond between Ala 587 and Ser 586 has the role of hydrogen bond donor to the oxygen of the methoxy group. This potential and probable hydrogen bonds maybe a logical reason for higher enzyme inhibitory potency of compound **4d** compared to others.

### Structure activity relationship

Enzyme inhibitory effect of the final prepared compounds was investigated towards 15-lipoxygense-1 and obtained results were listed in Table 2. None of the tested derivatives exerted favorable inhibitory potency towards 15-LOX-1 at 200 μM concentration compared to quercetin as reference compound. Quercetin as naturally occurring compound showed 100 % inhibition at 200 μM concentration. Among the tested derivatives, compound **4d** with *meta* positioning of the methoxy group afforded the highest inhibitory effect (38 %). Movement of the methoxy to the *para* position caused a detrimental effect and decreased the inhibitory activity. Nitro containing compounds (**4a, 4b, 4c**) as well as *para* chlorine containing compound (**4 k**) did not demonstrated any inhibitory activity at tested concentration. Fluorine containing derivatives also exhibited a high inhibitory activity compared to others. This inhibitory activity was more observable while the fluorine substituted at position *ortho* and *para*. It is probable that electron withdrawing effect of this moiety is a critical factor to increase the enzyme inhibitory effect. Replacement of the fluorine substituent with chlorine caused a decrease in activity. Chlorine atom has a greater size and lipophilicity than fluorine and it is likely that these parameters induce a negative effect on interaction of the ligands to the corresponding receptor.

Three cancerous cell lines were used to evaluate the *in vitro* cytotoxicity potency of synthesized derivatives. Unfortunately, tested compounds did not display a remarkable activity against utilized cell lines (Table 2). Only compounds **4f** (*ortho* fluorine) and **4 k** (*para* chlorine) rendered an acceptable cytotoxic potency against HT29 cell line. None of the nitro and methoxy substituted derivatives exerted an inhibitory potency (IC_50_) up to 100 μM concentration. Substitution of the fluorine at *meta* position of the phenyl residue caused an enhancement in cytotoxic activity towards SKNMC cell line. *Para* positioning of the fluorine was also caused a brief increase in anticancer activity in PC3 cell line. A descending trend in IC_50_ was seen from compound **4i** (*o*-Cl) to **4 k** (*p*-Cl) against PC3 cell line. But, a reverse trend was demonstrated for chlorinated derivatives against HT29 and SKNMC cell lines. Steric factor and lipophilicity effects produced by chlorine atom maybe a probable reason for this observation.

Based on the above mentioned information, there is not any direct correlation between the cytotoxic activity and inhibition of lipoxygenase. According to the previously reported literature [34, 35], it could be hypothesized that inhibition of tyrosine kinases is the probable of cytotoxicity of these compounds.

## Conclusions

A new series of compounds with combination of 1,3,4-thiadiazole and phthalimide substructures were synthesized and their cytotoxicity was evaluated *in vitro* using MTT protocol. Furthermore, synthesized derivatives were tested in an enzymatic assay for exploration of the inhibitory activity towards 15-lipoxygenase-1. According to the obtained results in MTT assay as well as enzymatic experiment, the investigated compounds did not show a favorable anticancer activity. Amongst them, only compounds **4f** (*ortho* fluorine) and **4 k** (*para* chlorine) exhibited an acceptable cytotoxic potency towards HT29 cell line. Hence, further structural modifications are needed to achieve derivatives with superior activity.

## References

[1] Rioux N, Castonguay A (1998). Inhibitors of lipoxygenase: a new class of cancer chemopreventive agents. Carcinogenesis.

[2] Tong WG, Ding XZ, Adrian TE (2002). The mechanisms of lipoxygenase inhibitor-induced apoptosis in human breast cancer cells. Biochem Biophys Res Commun.

[3] Steele VE, Holmes CV, Hawk ET, Kopelovich L, Lubet RA, Crowell JA (1999). Lipoxygenase inhibitors as potential cancer chemopreventives. Cancer Epidemiol Biomarkers Prev.

[4] Shureiqi I, Chen D, Jack Lee J, Yang P, Newman RA, Brenner DE (2000). 15-LOX-1: a novel molecular target of nonsteroidal anti-inflammatory drug-Induced apoptosis in colorectal cancer cells. J Natl Cancer Inst.

[5] Shureiqi I, Lippman SM (2001). Lipoxygenase modulation to reverse carcinogenesis. Cancer Res.

[6] Kelavkar UP, Nixon JB, Cohen C, Dillehay D, Eling TE, Badr KF (2001). Overexpression of 15-lipoxygenase-1 in PC-3 human prostate cancer cells increases tumorigenesis. Carcinogenesis.

[7] Shureiqi I, Xu X, Chen D, Lotan R, Morris JS, Fischer SM (2001). Nonsteroidal anti-Inflammatory drugs induce apoptosis in esophageal cancer cells by restoring 15-Lipoxygenase-1 expression. Cancer Res.

[8] Mahdavi M, Shirazi MS, Taherkhani R, Saeedi M, Alipour E, Moghadam FH (2014). Synthesis, biological evaluation and docking study of 3-aroyl-1-(4-sulfamoylphenyl)thiourea derivatives as 15-lipoxygenase inhibitors. Eur J Med Chem.

[9] Asadipour A, Edraki N, Nakhjiri M, Yahya-Meymandi A, Alipour E, Saniee P (2013). Anti-Helicobacter pylori activity and structure-activity relationship study of 2-Alkylthio-5-(nitroaryl)-1,3,4-thiadiazole derivatives. Iran J Pharm Res.

[10] Mohammadhosseini N, Saniee P, Ghamaripour A, Aryapour H, Afshar F, Edraki N (2013). Synthesis and biological evaluation of novel benzyl piperazine derivatives of 5-(5-nitroaryl)-1,3,4-thiadiazoles as Anti-Helicobacter pylori agents. Daru: J Pharm Sci.

[11] Moshafi MH, Sorkhi M, Emami S, Nakhjiri M, Yahya-Meymandi A, Negahbani AS (2011). 5-Nitroimidazole-based 1,3,4-thiadiazoles: heterocyclic analogs of metronidazole as anti-Helicobacter pylori agents. Arch Pharm Chem Life Sci.

[12] Rajak H, Deshmukh R, Aggarwal N, Kashaw S, Kharya MD, Mishra P (2009). Synthesis of novel 2,5-disubstituted 1,3,4-thiadiazoles for their potential anticonvulsant activity: pharmacophoric model studies. Arch Pharm Chem Life Sci.

[13] Abdel-Aziz M, Aly OM, Khan SS, Mukherjee K, Bane S (2012). Synthesis, Cytotoxic properties and tubulin polymerization inhibitory activity of novel 2-pyrazoline derivatives. Arch Pharm Chem Life Sci.

[14] Mishra G, Singh AK, Jyoti K (2011). Review article on 1,3,4-Thiadiazole derivatives and its pharmacological activities. Int J Chem Tech Res.

[15] Singh AK, Mishra G, Jyoti K (2011). Review on Biological Activities of 1,3,4-Thiadiazole derivatives. J App Pharm Sci.

[16] Kalidhar U, Kaur A (2011). 1,3,4-Thiadiazole derivatives and their biological activities: A Review. Res J Pharm Biol Chem.

[17] Siddiqui N, Ahuja P, Ahsan W, Pandeya SN, Alam MS (2009). Thiadiazoles: progress report on biological activities. J Chem Pharm Res.

[18] Behrouzi-Fardmoghadam M, Poorrajab F, Kaboudanian Ardestani S, Emami S, Shafiee A, Foroumadi A (2008). Synthesis and in vitro anti-leishmanial activity of 1-[5-(5-nitrofuran-2-yl)-1,3,4-thiadiazol-2-yl]- and 1-[5-(5-nitrothiophen-2-yl)-1,3,4-thiadiazol-2-yl]-4-aroylpiperazines. Bioorg Med Chem.

[19] Poorrajab F, Kaboudanian Ardestani S, Emami S, Behrouzi-Fardmoghadam M, Shafiee A, Foroumadi A (2009). Nitroimidazolyl-1,3,4-thiadiazole-based anti-leishmanial agents: Synthesis and in vitro biological evaluation. Eur J Med Chem.

[20] Mirzaei J, Siavoshi F, Emami S, Safari F, Khoshayand MR, Shafiee A (2008). Synthesis and in vitro anti-Helicobacter pylori activity of *N*-[5-(5-nitro-2-heteroaryl)-1,3,4-thiadiazol-2-yl]thiomorpholines and related compounds. Eur J Med Chem.

[21] Kok SHL, Gambari R, Chui CH, Yuen MCW, Lin E, Wong RSM (2008). Synthesis and anti-cancer activity of benzothiazole containing phthalimide on human carcinoma cell lines. Bioorg Med Chem.

[22] Yang YJ, Yang YN, Jiang JS, Feng ZM, Liu HY, Pan XD (2010). Synthesis and cytotoxic activity of heterocycle-substituted phthalimide derivatives. Chin Chem Lett.

[23] Machado AL, Lima LM, Araújo-Jr JX, Fraga CAM, Koatzc VLG, Barreiroa EJ (2005). Design, synthesis and antiinflammatory activity of novel phthalimide derivatives, structurally related to thalidomide. Bioorg Med Chem Lett.

[24] Malgorzata W, Katarzyna K (2009). Synthesis and anticonvulsant evaluation of some *N*-substituted phthalimides. Acta Pol Pharm.

[25] Lee N-J, Jeong I-C, Cho M-Y, Jeon C-W, Yun B-C, Kim Y-O (2006). Synthesis and *in vitro* antitumor activity of phthalimide polymers containing podophyllotoxin. Eur Polym J.

[26] Lima LM, Brito FCF, Souza SD, Miranda ALP, Rodrigues CR, Fragaa CAM (2002). Novel phthalimide derivatives, designed as leukotriene D_4_ receptor antagonists. Bioorg Med Chem Lett.

[27] Santos JL, Yamasaki PR, Chin CM, Takashi CH, Pavan FR, Leite CQF (2009). Synthesis and in vitro anti mycobacterium tuberculosis activity of a series of phthalimide derivatives. Bioorg Med Chem.

[28] Mohammadi-Farani A, Ahmadi A, Nadri H, Aliabadi A (2013). Synthesis, docking and acetylcholinesterase inhibitory assessment of 2-(2-(4-Benzylpiperazin-1-yl)ethyl)isoindoline-1,3-dione with potential anti-alzheimer effects. Daru J Pharm Sci.

[29] Foroumadi A, Mohammadi-Farani A, Garmsiri Mahvar M, Aliabadi A (2013). Synthesis and evaluation of anti-acetylcholinesterase activity of 2-(2-(4-(2-Oxo-2-phenylethyl)piperazin-1-yl)ethyl)isoindoline-1,3-dione derivatives with potential anti-Alzheimer effects. Iran J Basic Med Sci.

[30] Aliabadi A, Gholamine B, Karimi T (2014). Synthesis and antiseizure evaluation of isoindoline-1,3-dione derivatives in mice. Med Chem Res.

[31] Aliabadi A, Foroumadi A, Safavi M, Kaboudian Ardestani S (2012). Synthesis, molecular docking and cytotoxicity evaluation of 2-(4-substituted-benzyl)isoindoline-1,3-dione derivatives as anticancer agents. J Rep Pharm Sci.

[32] Ragavendran JV, Sriram D, Patel SK, Reddy IV, Bharathwajan N, Stables J (2007). Design and synthesis of anticonvulsants from a combined phthalimide-GABA-anilide and hydrazone pharmacophore. Eur J Med Chem.

[33] Mohammadi-Farani A, Foroumadi A, Rezvani Kashani M, Aliabadi A (2014). *N*-Phenyl-2-*p*-tolylthiazole-4-carboxamide derivatives: Synthesis and cytotoxicity evaluation as anticancer agents. Iran J Basic Med Sci.

[34] Aliabadi A, Eghbalian E, Kiani A (2013). Synthesis and cytotoxicity evaluation of a series of 1,3,4-thiadiazole based compounds as anticancer agents. Iran J Basic Med Sci.

[35] Aliabadi A, Hasanvand Z, Kiani A, Mirabdali SS (2013). Synthesis and *in vitro* cytotoxicity assessment of *N*-(5-(Benzylthio)-1,3,4-thiadiazol-2-yl)-2-(4-(trifluoromethyl)phenyl)acetamide with potential anticancer activity. Iran J Pharm Res.

[36] Anthon GE, Barrett DM (2001). Colorimetric method for the determination of lipoxygenase activity. J Agric Food Chem.

[37] ArgusLab 4.0 Mark A. Thompson Planaria Software LLC, Seattle, WA http://www.arguslab.com.

